# Clinical impact of extensive molecular profiling in advanced cancer patients

**DOI:** 10.1186/s13045-017-0411-5

**Published:** 2017-02-08

**Authors:** Sophie Cousin, Thomas Grellety, Maud Toulmonde, Céline Auzanneau, Emmanuel Khalifa, Yec’han Laizet, Kevin Tran, Sylvestre Le Moulec, Anne Floquet, Delphine Garbay, Jacques Robert, Isabelle Hostein, Isabelle Soubeyran, Antoine Italiano

**Affiliations:** 1 Early Phase Trials Unit, Institut Bergonié, 229 Cours de l’Argonne, 33000 Bordeaux, France; 2 Department of Medicine, Institut Bergonié, 229 Cours de l’Argonne, 33000 Bordeaux, France; 3 Department of Biopathology, Institut Bergonié, 229 Cours de l’Argonne, 33000 Bordeaux, France; 4 Department of Bioinformatics, Institut Bergonié, 229 Cours de l’Argonne, 33000 Bordeaux, France

## Abstract

Previous precision medicine studies have investigated conventional molecular techniques and/or limited sets of gene alterations. The aim of this study was to describe the impact of the next-generation sequencing of the largest panel of genes used to date in tumour tissue and blood in the context of institutional molecular screening programmes. DNA analysis was performed by next-generation sequencing using a panel of 426 cancer-related genes and by comparative genomic hybridization from formalin-fixed and paraffin-embedded archived tumour samples when available or from fresh tumour samples. Five hundred sixty-eight patients were enrolled. The median number of prior lines of treatment was 2 (range 0–9). The most common primary tumour types were lung (16.9%), colorectal (14.4%), breast (10.6%), ovarian (10.2%) and sarcoma (10.2%). The median patient age was 63 years (range 19–88). A total of 292 patients (51.4%) presented with at least one actionable genetic alteration. The 20 genes most frequently altered were *TP53*, *CDKN2A*, *KRAS*, *PTEN*, *PI3KCA*, *RB1*, *APC*, *ERBB2*, *MYC*, *EGFR*, *CDKN2B*, *ARID1A*, *SMAD4*, *FGFR1*, *MDM2*, *BRAF*, *ATM*, *CCNE1*, *FGFR3* and *FRS2*. One hundred fifty-nine patients (28%) were included in early phase trials. The treatment was matched with a tumour profile in 86 cases (15%). The two main reasons for non-inclusion were non-progressive disease (31.5%) and general status deterioration (25%). Twenty-eight percent of patients presented with a growth modulation index (time to progression under the early phase trial treatment/time to progression of the previous line of treatment) >1.3.

Extensive molecular profiling using high-throughput techniques allows for the identification of actionable mutations in the majority of cases and is associated with substantial clinical benefit in up to one in four patients.

**Electronic supplementary material:**

The online version of this article (doi:10.1186/s13045-017-0411-5) contains supplementary material, which is available to authorized users.

## Letter to the editor

Previous precision medicine studies have investigated conventional molecular techniques and/or limited sets of gene alterations [1–3]. We describe here the impact of the next-generation sequencing of the largest panel of genes used to date in tumour tissue and blood in the context of institutional molecular screening programmes. The eligibility criteria, methods of sequencing and statistics are described in Additional file 1.

Between January 1, 2014, and June 30, 2015, 568 patients were enrolled in the study. Their characteristics are summarized in Additional file 2: Table S1 and Additional file 3: Figure S1.

In 28 cases (5%), molecular analysis failed mainly because of insufficient tissue quantity or quality. The median time from first referral to reporting was 9 weeks (range 1–36 weeks). The 20 genes found most frequently altered were *TP53*, *CDKN2A*, *KRAS*, *PTEN*, *PI3KCA*, *RB1*, *APC*, *ERBB2*, *MYC*, *EGFR*, *CDKN2B*, *ARID1A*, *SMAD4*, *FGFR1*, *MDM2*, *BRAF*, *ATM*, *CCNE1*, *FGFR3* and *FRS2* (Additional file 4: Figure S2). One thousand and six hundred fifty-nine alterations were found: 883 mutations (53.2%), 755 (45.5%) gene copy number alterations and 21 (1.3%) fusions. The median number of alterations per patient was 2 (range 0–18).

Two hundred ninety-two (51.4%) patients had at least one genetic alteration that was considered actionable by the molecular tumour board. Molecular profiles by tumour type are presented in Additional file 5: Figure S3.

**Fig. 1:**
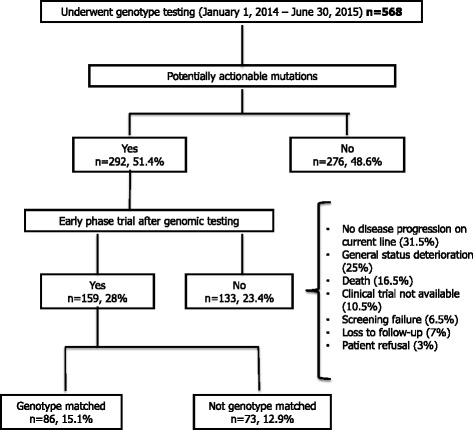
Study flow diagram

One hundred fifty-nine (28%) patients were randomized in an early phase clinical trial (EPCT) after the screening results. The main reasons for non-inclusion were non-progressive disease on current treatment regimen (31.5%), general status deterioration (25%), death (16.5%), clinical trial not available (10.5%), screening failure (6.5%), loss to follow-up (7%) and patient refusal (3%). The drug used in the EPCT was genotype-matched (GM) in 86 (15.1%) patients and non-matched (NM) in 73 (12.9%) patients (Fig. 1). The drugs received are summarized in Additional file 2: Table S2.

In the GM group, 65 patients were evaluable for the response to treatment analysis. The disease control rate (objective response rate + stable disease) was 47.7% (Additional file 2: Table S3). The median progression-free survival (PFS) was 3 months. The median overall survival was 8.5 months (range 5.5–11.5 months).

Fifty-nine patients were evaluable for the growth modulatin index (GMI) calculation. The median GMI [4] was 0.63 (0.01–5.81). Twenty patients (27.8%) had a GMI ≥ 1.3 (Additional file 2: Table S3). A GMI ≥ 1.3 was associated with a trend towards improved median overall survival: 11.7 months (range 0.3–23.1) versus 7.6 months (range 4.8–10.5 months) for GMI < 1.3, which was not statistically significant (*p* = 0.28).

Thirty-nine patients with coupled primary and metastatic tumours were analysed to evaluate the correlation between the molecular screening results of the two samples. Twenty-six patients (67%) had at least one mutation considered targetable. In this population, 9 cases had a discordant mutational status between the primary and metastatic sites. This discordance was related to an actionable mutation in only four cases for a final concordance rate in terms of targetable alterations of 85% (22/26 patients).

Seventy-five patients underwent also a tumour molecular profile-based circulating-free DNA (cfDNA) analysis. Their characteristics are shown in Additional file 2: Table S4. Ninety-five genetic aberrations were found: 86 (90.5%) mutations, 7 (7.4%) gene copy number alterations and 2 (2.1%) fusions. Thirty-four (45.3%) patients were found to have at least one targetable mutation (median number 1; range 0–5). The most frequently altered genes are shown in Additional file 6: Figure S4. Ten patients (13.3%) were included in an EPCT, six (8%) of whom were included based on their tumour genotype profiles.

Our extensive molecular screening program allowed the identification of at least one actionable genetic alteration in 51.4% of cases and was associated with a significant clinical benefit since 27.8% of the patients in the GM group experienced a GMI > 1.3 (Additional file 2: Table S3). The low rate of technical failure and the high correlation rate between primary tumours and metastases demonstrate that FFPE archival tissue could be used effectively for molecular screening, making the need for invasive, resource-consuming and expensive tumour biopsies unnecessary. Due to tumour heterogeneity, biopsies often suffer from sample bias and archival tissue is not always available. In this regard, we report here for the first time the value of an NGS assay targeting 20 cancer genes to detect actionable mutations and rearrangements in cfDNA in the context of a precision medicine study.

Overall, this study demonstrates the feasibility and potentially positive clinical impact of using comprehensive molecular profiling to improve the outcomes of cancer patients.

## Additional files

Additional file 1: Supplementary Methods. (DOCX 14 kb)
Additional file 2: Table S1. Patient characteristics (*n* = 568). **Table S2.** Summary of the drugs received by the patients included in early phase trials (*n* = 159). **Table S3.** Tumour response rate and growth modulation index (GMI) value in patients with a matched treatment. **Table S4** Characteristics of the patients who underwent molecular screening on cell-free plasma DNA (*n* = 75). **Table S5.** Four hundred twenty-six genes screened for base substitutions, insertion-deletions, copy number changes and rearrangements. **Table S6.** List of cancer-related genes identified in cell-free plasma DNA samples. (DOCX 45 kb)
Additional file 3: Figure S1. Distribution of the cancer types tested for molecular screening. (DOCX 16 kb)
Additional file 4: Figure S2. Most frequently altered genes identified by molecular screening. (DOCX 17 kb)
Additional file 5: Figure S3. Molecular profiles of the ten most frequent tumour types. (PPTX 62 kb)
Additional file 6: Figure S4. The most frequently altered genes in cell-free plasma DNA samples. (DOCX 18 kb)
