# Supplementary material for: Clinical impact of extensive molecular profiling in advanced cancer patients
Source: J Hematol Oncol. 2017 Feb 8;10:45. doi: 10.1186/s13045-017-0411-5 (PMC5299780; doi:10.1186/s13045-017-0411-5)
Supplement: Additional file 1: — Supplementary Methods. (DOCX 14 kb) [file 13045_2017_411_MOESM1_ESM.docx]

**Patients**

Eligibility criteria were as follows: patients with solid tumours, > 18 years old, Eastern Cooperative Oncology Group performance status < 2, and measurable disease according to Response Evaluation Criteria in Solid Tumors (RECIST) version 1.1. Patients were required to have adequate amounts of absolute neutrophils (>1x10^9^ cells/L), platelets (>1x10^11^ cells/L), haemoglobin (>90 g/L), serum creatinine (<1·5 times the upper limit of normal (ULN)), serum total bilirubin (<1·5 times ULN), and aspartate and alanine aminotransferases (<3 times the ULN or <5 times the ULN for patients with liver metastasis). The exclusion criteria were as follows: prior therapy-related toxicities of grades >2 according to the NCI-CTCAE v4.0 and progressive leptomeningeal or brain metastases. Patients with brain metastases that were locally treated and stable for more than 1 month were eligible for inclusion.

The protocol was approved by the Institutional review Board of Institut Bergonié national ethics committee and was performed in accordance to the Good Clinical Practice guidelines of the International Conference on Harmonization.

**Methods**

To analyse the tumour molecular profile, tumour DNA was isolated from a formalin-fixed and paraffin-embedded (FFPE) archived sample when available or from a fresh tumour biopsy when an FFPE specimen was not available. Massive parallel DNA sequencing to characterize base substitutions, short insertions and deletions (indels), copy number alterations, and selected fusions across 426 cancer-related genes (**Suppl. Table 5**) was performed as previously described.^1^ Moreover, comprehensive analysis of gene copy number alterations was performed using comparative genomic hybridization, as previously described.^2^ The patients were also assessed for biomarkers according to the standard of care, including HER2 status by immunohistochemistry in breast and gastric cancer and oestrogen and progesterone receptor expression in breast cancer.

A genetic alteration was considered actionable when one of the following criteria were met: (i) directly targetable by small molecule inhibitors or a therapeutic antibody; or (ii) indirectly targetable by a therapeutic agent impacting ≥ 1 effector in the signalling pathway.

The results for each patient were discussed by a weekly multidisciplinary molecular tumour board to assess their eligibility for an early phase clinical trial based on the screening results. In patients in whom no targetable alterations were identified, and when possible (trial available and patient eligible), patients were directed to a non-matched early phase clinical trial.

When available, molecular screening was performed on the primary tumour sample and on a metastasis in the same patient to evaluate the degrees of concordance and discordance for the genetic alterations identified.

Seventy-five patients also benefitted from a pilot sub-study investigating the use of a cell-free plasma DNA (cfDNA) assay as an alternative to tumour tissue sample genetic analysis, as previously described.^7^ A list of the alterations screened is provided in **Suppl. Table 6**.

**Statistical analysis**

The description of the patients was based on classical descriptive statistics: number of cases, percentages, means and standard deviations, medians, and extreme values. Categorical variables were summarized in frequency tables. Response rate was evaluated using RECIST 1.1. The Kaplan-Meier method and the log-rank test were used to analyse the survival data. The growth modulation index (GMI) was calculated, which is defined as the ratio of the time to progression (TTP) under the early phase clinical trial treatment (treatment N) to the TTP under the previous line of treatment (treatment N-1): GMI=^TTP(N)^/_TTP(N-1)_.^3^ The GMI was used to allow for intra-patient comparisons, which is thought to be relevant for exploring the benefit of a screening-based personalized anti-cancer treatment, relative to previously received treatments. A GMI cut-off > 1.3 is believed to demonstrate a non-ambiguous sign of activity for drug N.^4,5^

**References**:

1. Frampton GM, Fichtenholtz A, Otto GA, et al. Development and validation of a clinical cancer genomic profiling test based on massively parallel DNA sequencing. *Nat Biotechnol* 2013; **31**: 1023–31.

2. Italiano A, Lagarde P, Brulard C, et al. Genetic profiling identifies two classes of soft-tissue leiomyosarcomas with distinct clinical characteristics. *Clin Cancer Res* 2013; **19**: 1190–6.

3. Von Hoff DD. There are no bad anticancer agents, only bad clinical trial designs–twenty-first Richard and Hinda Rosenthal Foundation Award Lecture. *Clin Cancer Res* 1998; **4**: 1079–86.

4. Cousin S, Blay JY, Bertucci F, et al. Correlation between overall survival and growth modulation index in pre-treated sarcoma patients: a study from the French Sarcoma Group. *Ann Oncol* 2013; **24**: 2681–5.

5. Von Hoff DD, Stephenson JJ Jr, Rosen P, et al. Pilot study using molecular profiling of patients’ tumors to find potential targets and select treatments for their refractory cancers. *J Clin Oncol* 2010; **28**: 4877–83.
