# Supplementary material for: Clinical impact of extensive molecular profiling in advanced cancer patients
Source: J Hematol Oncol. 2017 Feb 8;10:45. doi: 10.1186/s13045-017-0411-5 (PMC5299780; doi:10.1186/s13045-017-0411-5)
Supplement: Additional file 2: Table S1. — Patient characteristics (n = 568). Table S2. Summary of the drugs received by the patients included in early phase trials (n = 159). Table S3. Tumour response rate and growth modulation index (GMI) value in patients with a matched treatment. Table S4 Characteristics of the patients who underwent molecular screening on cell-free plasma DNA (n = 75). Table S5. Four hundred twenty-six genes screened for base substitutions, insertion-deletions, copy number changes and rearrangements. Table S6. List of cancer-related genes identified in cell-free plasma DNA samples. (DOCX 45 kb) [file 13045_2017_411_MOESM2_ESM.docx]

**Supplementary Table 1: Patient characteristics (n=568)**

|  | | N | % |
| --- | --- | --- | --- |
| Sex | Male  Female | 247  321 | 43.5  56.5 |
| Age (years) | Median: 63 [range: 19 – 88] | | |
| Performance status (ECOG) | 0  1  2 | 219  316  33 | 38.6  55.6  5.8 |
| Tumour type | Lung  Colorectal  Breast  Ovarian  Sarcoma  Other | 96  82  60  58  58  214 | 16.9  14.4  10.6  10.2  10.2  37.5 |
| Previous lines | Median: 2 [range: 0-9] | | |
| Progressive disease at screening | Yes  No | 299  269 | 52.6  47.4 |

**Supplementary Table 2: Summary of the drugs received by the patients included in early phase trials (n=159)**

| **Mechanism of action** | **Type of molecule** | **N (%)** |
| --- | --- | --- |
| **MATCHED TREATMENT (N=86)** | | |
| *MAP KINASE PATHWAY:* | | |
| RAF inhibitor | Small molecule | 4 (2·5) |
| RAF and MEK inhibitor | Small molecules | 1 (0·6) |
| *AKT/MTOR PATHWAY:* | | |
| PI3KCA inhibitor | Small molecule | 1 (0·6) |
| p70/AKT inhibitor | Small molecule | 10 (6) |
| MTOR inhibitor | Small molecule | 18 (11·3) |
| *TYROSINE KINASE INHIBITORS* | | |
| EGFR inhibitor | Small molecule | 6 (3·8) |
| HER2 inhibitor | Small molecule | 7 (4·4) |
| FGFR inhibitor | Pan FGFR inhibitor | 13 (8·2) |
| Multi TKI inhibitor | Small molecule | 25 (15·7) |
| *OTHER* |  |  |
| Inhibitor of p53: MDM2 interaction | Small molecule | 1 (0·6) |
| **UNMATCHED TREATMENT (N=73)** | | |
| *IMMUNOTHERAPIES/Immunomodulation:* | | |
| PD-1/PD-L1 pathway | Monoclonal antibodies | 10 (6) |
| CSF-1R/CSF1 | Monoclonal antibody | 23 (14·5) |
| Oncolytic virus | Vaccine | 1 (0·6) |
| Immunomodulation | Metronomic chemotherapy | 5 (3·1) |
| *EPIGENETIC:* | | |
| Bromodomain inhibitor | Small molecule | 1 (0·6) |
| EZH2 inhibitor | Histone-lysine methyltransferase EZH2 gene inhibitor | 7 (4·4) |
| *CELL CYCLE INHIBITION:* | | |
| Checkpoint kinase 1 inhibitor  CDK4/6-Cyclin D1 inhibitor | Small molecule  Small molecule | 9 (5·7)  1 (0·6) |
| *OTHERS:* | | |
| Hormonotherapy | Progesterone receptor inhibitor | 12 (7·5) |
| Antiviral drug | Non-nucleoside reverse transcriptase inhibitor | 1 (0·6) |
| DNA damage reparation | PARP inhibitor | 3 (1·9) |
|  | **N total** | **159 (100)** |

**Supplementary Table 3: Tumour response rate and Growth Modulation Index (GMI) value in patients with a matched treatment**

| Response Rate according to RECIST 1.1 (n=65) | |
| --- | --- |
| Treatment | Matched to molecular alteration (n=65) |
|  | N (%) |
| Complete response* | 0 (0) |
| Partial response* | 7 (10.8) |
| Stable disease* | 24 (36.9) |
| Progressive disease* | 34 (52.3) |
| Growth Modulation Index (n=72) | |
|  | N (%) |
| GMI <1.33 | 52 (72.2) |
| GMI > 1.33 | 20 (27.8) |

**Supplementary Table 4: Characteristics of the patients who underwent molecular screening on cell-free plasma DNA (n=75)**

|  | | N | % |
| --- | --- | --- | --- |
| Sex | Male  Female | 33  42 | 44  56 |
| Age (years) | Median: 62 [range: 24 - 85] | | |
| Tumour type | Lung  Breast  Ovarian  Head and neck  Biliary tract  Urothelial  Other | 53  5  5  4  3  2  3 | 70·7  6·7  6·7  5·3  4  2·6  4 |
| Previous lines | Median: 2 [range: 0 - 8] | | |

**Supplementary Table 5: 426 genes screened for base substitutions, insertion-deletions, copy number changes and rearrangements**

**395 genes screened for base substitutions, insertion-deletions and copy number changes.**

*Promoter region only.

| ABL1  ABL2  ACVR1B AKT1  AKT2  AKT3  ALK ALOX12B AMER1 | CCND2  CCND3  CCNE1  CD274  CD79A CD79B CDC73  CDH1  CDH2 | EPHA5  EPHA6  EPHA7  EPHB1  EPHB4  EPHB6  ERBB2  ERBB3  ERBB4 | FUBP1  GABRA6  GALNT12  GATA1  GATA2  GATA3  GATA4  GATA6  GEN1 | KDM5A KDM5C KDM6A KDR KEAP1  KEL KIT KLHL6  KMT2A (MLL) | NF2  NFE2L2  NFKBIA NKX2-1  NOTCH1  NOTCH2  NOTCH3  NOTCH4  NPM1 | PRKDC PRSS1  PRSS8  PTCH1  PTCH2  PTEN PTPN11  PTPRD QKI | SOX9  SPEN SPOP SPTA1  SRC STAG2  STAT3  STAT4  STK11 |
| --- | --- | --- | --- | --- | --- | --- | --- |
| (FAM123B)  APC | CDH20 | ERCC4 | GID4 (C17orf39) | KMT2C (MLL3) | NRAS | RAC1 | SUFU |
| APCDD1 | CDH5 | ERG | GLI1 | KMT2D (MLL2) | NSD1 | RAD50 | SYK |
| AR | CDK12 | ERRFI1 | GNA11 | KRAS | NTRK1 | RAD51 | TAF1 |
| ARAF | CDK4 | ESR1 | GNA13 | LMO1 | NTRK2 | RAD51B (RAD51L1) | TBX3 |
| ARFRP1 | CDK6 | EZH2 | GNAQ | LRP1B | NTRK3 | RAD51C | TEK |
| ARID1A | CDK8 | FAM175A | GNAS | LRP6 | NUDT1 | RAD51D (RAD51L3) | TERC |
| ARID1B  ARID2 | CDKN1A  CDKN1B | FAM46C  FANCA | GPR124  GREM1 | LTK  LYN | NUP93  PAK3 | RAD52  RAD54L | TERT (promoter  only)  TET2 |
| ASXL1 | CDKN2A | FANCC | GRIN2A | LZTR1 | PAK7 | RAF1 | TGFBR2 |
| ATM | CDKN2B | FANCD2 | GRM3 | MAGI2 | PALB2 | RANBP2 | TIPARP |
| ATR | CDKN2C | FANCE | GSK3B | MAP2K1 | PARK2 | RARA | TNF |
| ATRX | CEBPA | FANCF | H3F3A | MAP2K2 | PARP1 | RB1 | TNFAIP3 |
| AURKA | CHD2 | FANCG | HGF | MAP2K4 | PARP2 | RBM10 | TNFRSF14 |
| AURKB | CHD4 | FANCI | HLA-A | MAP3K1 | PARP3 | REL | TNKS |
| AXIN1 | CHEK1 | FANCL | HLA-B | MAP3K13 | PARP4 | RET | TNKS2 |
| AXL | CHEK2 | FANCM | HLA-C | MCL1 | PAX5 | RICTOR | TOP1 |
| BACH1 | CHUK | FAS | HNF1A | MDM2 | PBRM1 | RNF43 | TOP2A |
| BAP1 | CIC | FAT1 | HOXB13 | MDM4 | PDCD1LG2 | ROS1 | TP53 |
| BARD1 | CRBN | FAT3 | HRAS | MED12 | PDGFRA | RPA1 | TP53BP1 |
| BCL2 | CREBBP | FBXW7 | HSD3B1 | MEF2B | PDGFRB | RPTOR | TRRAP |
| BCL2A1 | CRKL | FGF10 | HSP90AA1 | MEN1 | PDK1 | RUNX1 | TSC1 |
| BCL2L1 | CRLF2 | FGF12 | IDH1 | MERTK | PHLPP2 | RUNX1T1 | TSC2 |
| BCL2L2 | CSF1R | FGF14 | IDH2 | MET | PIK3C2B | SDHA | TSHR |
| BCL6 | CTCF | FGF19 | IGF1 | MITF | PIK3C2G | SDHB | TYRO3 |
| BCOR | CTNNA1 | FGF23 | IGF1R | MKNK1 | PIK3C3 | SDHC | U2AF1 |
| BCORL1 | CTNNB1 | FGF3 | IGF2 | MKNK2 | PIK3CA | SDHD | VEGFA |
| BLM | CUL3 | FGF4 | IGF2R | MLH1 | PIK3CB | SETD2 | VHL |
| BMPR1A | CUL4A | FGF6 | IKBKE | MPL | PIK3CG | SF3B1 | WISP3 |
| BRAF | CUL4B | FGF7 | IKZF1 | MRE11A | PIK3R1 | SH2B3 | WT1 |
| BRCA1 | CYLD | FGFR1 | IL7R | MSH2 | PIK3R2 | SLIT2 | XPO1 |
| BRCA2 | CYP17A1 | FGFR2 | INHBA | MSH6 | PLCG2 | SMAD2 | XRCC2 |
| BRD4 | DAXX | FGFR3 | INPP4B | MST1R | PMS2 | SMAD3 | XRCC3 |
| BRIP1 | DDR1 | FGFR4 | INSR | MTOR | PNRC1 | SMAD4 | ZBTB2 |
| BTG1 | DDR2 | FH | IRF2 | MUTYH | POLD1 | SMARCA4 | ZNF217 |
| BTK | DICER1 | FLCN | IRF4 | MYC | POLE | SMARCB1 | ZNF703 |
| C11orf30 (EMSY) | DIS3 | FLT1 | IRS2 | MYCL (MYCL1) | PPARG | SMARCD1 | ZNRF3 |

| CARD11 | DNMT3A | FLT3 | JAK1 | MYCN | PPP2R1A | SMO |  |
| --- | --- | --- | --- | --- | --- | --- | --- |
| CASP8 | DOT1L | FLT4 | JAK2 | MYD88 | PRDM1 | SNCAIP |  |
| CBFB | EGFR | FOXL2 | JAK3 | NBN | PREX2 | SOCS1 |  |
| CBL | EP300 | FOXP1 | JUN | NCOR1 | PRKAR1A | SOX10 |  |
| CCND1 | EPHA3 | FRS2 | KAT6A (MYST3) | NF1 | PRKCI | SOX2 |  |
| **Introns of 31 genes involved in rearrangements.** | | | | | | |  |
| ALK | BRCA1 | ETV1 | EWSR1 | KIT | MYC | PDGFRA | ROS1 |
| BCL2 | BRCA2 | ETV4 | FGFR1 | KMT2A (MLL) | NOTCH2 | RAF1 | RSPO2 |
| BCR | BRD4 | ETV5 | FGFR2 | MSH2 | NTRK1 | RARA | TMPRSS2 |
| BRAF | EGFR | ETV6 | FGFR3 | MYB | NTRK2 | RET |  |

**Supplementary Table 6: List of cancer-related genes identified in cell-free plasma DNA samples**

| **SNV/Indel** | **Fusions** | **CNV** | **Suppressors** |
| --- | --- | --- | --- |
| AKT1 ALK BRAF EGFR HER2 (ERBB2) KRAS NRAS MAP2K1 (MEK1) MET MET exon 14 skipping PIK3CA PTEN RET ROS1 | ALK FGFR3 NTRK1 RET ROS1 | EGFR FGFR1 HER2 (ERBB2) MET RICTOR | KEAP1 STK11 TP53 |
