# Supplementary material for: Clinical impact of extensive molecular profiling in advanced cancer patients
Source: J Hematol Oncol. 2017 Feb 8;10:45. doi: 10.1186/s13045-017-0411-5 (PMC5299780; doi:10.1186/s13045-017-0411-5)
Supplement: Additional file 3: Figure S1. — Distribution of the cancer types tested for molecular screening. (DOCX 16 kb) [file 13045_2017_411_MOESM3_ESM.docx]

**Figure 1: Distribution of the cancer types tested for molecular screening**
