# Supplementary material for: Clinical impact of extensive molecular profiling in advanced cancer patients
Source: J Hematol Oncol. 2017 Feb 8;10:45. doi: 10.1186/s13045-017-0411-5 (PMC5299780; doi:10.1186/s13045-017-0411-5)
Supplement: Additional file 4: Figure S2. — Most frequently altered genes identified by molecular screening. (DOCX 17 kb) [file 13045_2017_411_MOESM4_ESM.docx]

**Figure 2: Most frequently altered genes identified by molecular screening**
