# Supplementary material for: Clinical impact of extensive molecular profiling in advanced cancer patients
Source: J Hematol Oncol. 2017 Feb 8;10:45. doi: 10.1186/s13045-017-0411-5 (PMC5299780; doi:10.1186/s13045-017-0411-5)
Supplement: Additional file 5: Figure S3. — Molecular profiles of the ten most frequent tumour types. (PPTX 62 kb) [file 13045_2017_411_MOESM5_ESM.pptx]

## Slide 1
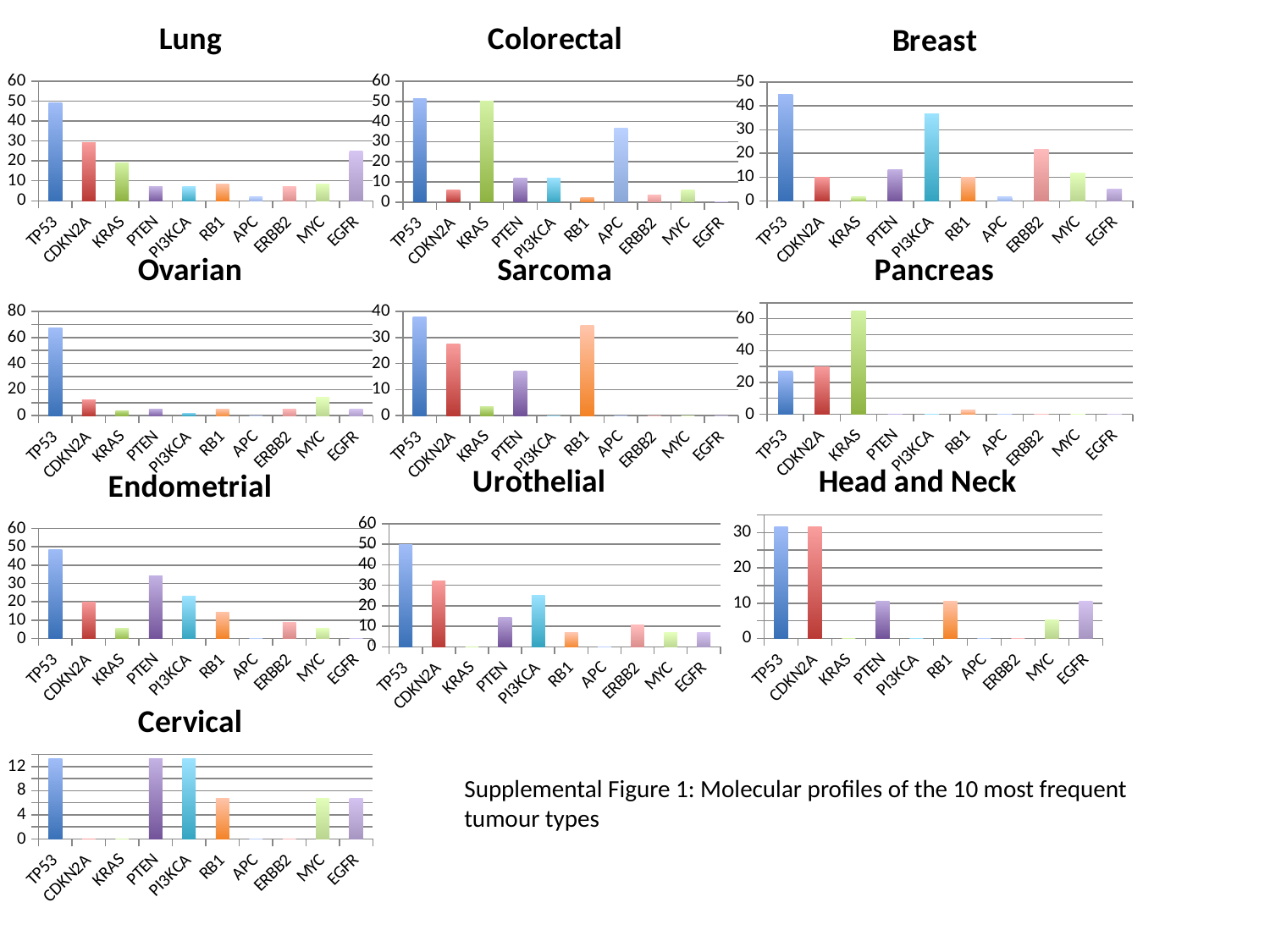

### Chart:
| Category | Lung |
|---|---|
| TP53 | 49.0 |
| CDKN2A | 29.2 |
| KRAS | 18.8 |
| PTEN | 7.3 |
| PI3KCA | 7.3 |
| RB1 | 8.3 |
| APC | 2.1 |
| ERBB2 | 7.3 |
| MYC | 8.3 |
| EGFR | 25.0 |
### Chart:
| Category | Colorectal |
|---|---|
| TP53 | 51.2 |
| CDKN2A | 6.1 |
| KRAS | 50.0 |
| PTEN | 12.1 |
| PI3KCA | 12.1 |
| RB1 | 2.4 |
| APC | 36.6 |
| ERBB2 | 3.7 |
| MYC | 6.1 |
| EGFR | 0.0 |
### Chart:
| Category | Breast |
|---|---|
| TP53 | 45.0 |
| CDKN2A | 10.0 |
| KRAS | 1.7 |
| PTEN | 13.3 |
| PI3KCA | 36.7 |
| RB1 | 10.0 |
| APC | 1.7 |
| ERBB2 | 21.7 |
| MYC | 11.7 |
| EGFR | 5.0 |
### Chart:
| Category | Ovarian |
|---|---|
| TP53 | 67.2 |
| CDKN2A | 12.1 |
| KRAS | 3.4 |
| PTEN | 5.2 |
| PI3KCA | 1.7 |
| RB1 | 5.2 |
| APC | 0.0 |
| ERBB2 | 5.2 |
| MYC | 13.8 |
| EGFR | 5.0 |
### Chart:
| Category | Sarcoma |
|---|---|
| TP53 | 37.9 |
| CDKN2A | 27.6 |
| KRAS | 3.4 |
| PTEN | 17.2 |
| PI3KCA | 0.0 |
| RB1 | 34.5 |
| APC | 0.0 |
| ERBB2 | 0.0 |
| MYC | 0.0 |
| EGFR | 0.0 |
### Chart:
| Category | Pancreas |
|---|---|
| TP53 | 27.0 |
| CDKN2A | 29.7 |
| KRAS | 64.9 |
| PTEN | 0.0 |
| PI3KCA | 0.0 |
| RB1 | 2.7 |
| APC | 0.0 |
| ERBB2 | 0.0 |
| MYC | 0.0 |
| EGFR | 0.0 |
### Chart:
| Category | Urothelial |
|---|---|
| TP53 | 50.0 |
| CDKN2A | 32.1 |
| KRAS | 0.0 |
| PTEN | 14.3 |
| PI3KCA | 25.0 |
| RB1 | 7.1 |
| APC | 0.0 |
| ERBB2 | 10.7 |
| MYC | 7.1 |
| EGFR | 7.1 |
### Chart:
| Category | Head and Neck |
|---|---|
| TP53 | 31.6 |
| CDKN2A | 31.6 |
| KRAS | 0.0 |
| PTEN | 10.5 |
| PI3KCA | 0.0 |
| RB1 | 10.5 |
| APC | 0.0 |
| ERBB2 | 0.0 |
| MYC | 5.3 |
| EGFR | 10.5 |
### Chart:
| Category | Endometrial |
|---|---|
| TP53 | 48.6 |
| CDKN2A | 20.0 |
| KRAS | 5.7 |
| PTEN | 34.2 |
| PI3KCA | 22.9 |
| RB1 | 14.3 |
| APC | 0.0 |
| ERBB2 | 8.6 |
| MYC | 5.7 |
| EGFR | 0.0 |
### Chart: Cervical
| Category | Cervix |
|---|---|
| TP53 | 13.3 |
| CDKN2A | 0.0 |
| KRAS | 0.0 |
| PTEN | 13.3 |
| PI3KCA | 13.3 |
| RB1 | 6.7 |
| APC | 0.0 |
| ERBB2 | 0.0 |
| MYC | 6.7 |
| EGFR | 6.7 |Supplemental Figure 1: Molecular profiles of the 10 most frequent tumour types
